# Gamma Irradiated *Pasteurella multocida* Vaccine induces strong humoral immunity and protects rabbits from disease

**DOI:** 10.1007/s11259-024-10388-y

**Published:** 2024-05-06

**Authors:** Sahar Ahmed, Waleed Abdelgaber Nemr, Asmaa El-Shershaby, Ehab Ali Mohamed Fouad, Mohamed Abd El-Fatah Mahmoud, Fatima Liaqat, Viskam Wijewardana, Hermann Unger

**Affiliations:** 1 Department of Cell Biology, Biotechnology Research Institute, National Research Centre, Giza, Egypt; 2 Department of Radiation Microbiology, National Centre for Radiation Research and Technology, Egyptian Atomic Energy Authority, Cairo, Egypt; 3 Department of Molecular Biology, Biotechnology Research Institute, National Research Centre, Giza, Egypt; 4 Department of Zoonosis, Veterinary Research Institute, National Research Centre, Giza, Egypt; 5 Department of Parasitology and Animal Diseases, Veterinary Research Institute, National Research Centre, Giza, Egypt; 6 Animal Production and Health Laboratory, Department of Nuclear Sciences and Applications, Joint FAO, IAEA Centre of Nuclear Techniques in Food and Agriculture, International Atomic Energy Agency, Vienna, Austria

**Keywords:** *Pasteurella multocida*, Gamma Irradiated Vaccine, ELISA, Serum IgG, Interferon Gamma

## Abstract

*Pasteurella multocida* is affecting a multitude of animals and severely affects livestock production. Existing vaccines are mostly chemically inactivated and do not lead to wide protection. Irradiated vaccines are enjoying a renaissance and the concept of “replication defficient but metabolically active” vaccines was recently evaluated in several vaccine trials. *P. multocida* was isolated from the nasal swab, blood, and lung swab samples from infected rabbits. Gamma irradiation of *P. multocida* for inhibition of replication was evaluated at an optimized irradiation dose of 10 Kgy established. Four groups of rabbits were (mock) vaccinated with a commercial *P. multocida* vaccine and three irradiated formulations as liquid, lyophilized formulations with added Trehalose and lyophilized-Trehalose with an “activation” culturing the irradiated bacteria for 24 in broth. Evaluation of humoral immune response by ELISA showed that all three irradiated vaccines produced an effective, protective, and continued IgG serum level after vaccination and bacterial challenge. The IFN-γ expression is maintained at a normal level, within each individual group however, the lyophilized trehalose irradiated vaccine showed peak mean of IFN-γ titer at one week after booster dose (day 21) which was statistically significant. Cumulatively, the results of this study show that gamma-irradiated *P. multocida* vaccines are safe and protect rabbits against disease. Moreover, Rabbits’ immunization with the three irradiated formulations avoided adverse side effects as compared to commercial polyvalent vaccine, the body weight gain for the irradiated vaccine groups indicates less stress compared to the commercial polyvalent vaccine.

## Introduction

Pasteurellosis i.e., infection commonly with *Pasteurella multocida* (*P. multocida*) contributes to significant production loss in animal farming and economic losses. It shows a high disease burden in wide range of domestic and wild animals such as cattle, pigs, rabbits, and chicken. This pathogen is mainly known for manifestation of fowl cholera in poultry, as well as bovine hemorrhagic septicemia in cattle, buffalo and camels, and snuffles in rabbits (Songer and Post 2004; Premalatha et al. 2009; Lennox 2012; Wilson and Ho 2013; WOAH 2020; Zhu et al. 2020; Mahrous et al. 2022). Evidence suggests that rabbits are infected soon after birth and they may carry this pathogen without any clinical signs (asymptomatic) for a long period of time (Palócz et al. 2014). Prevalence rate of 47.7% of *P. multocida* was reported from an Egyptian chicken farm (Ismail and Eid 2022).

*P. multocida* is known to be highly contagious, with animal transmission mainly by inhalation of aerosols, ingestion of contaminated feed, water, soil or infected carcasses (WOAH 2020). It is an opportunistic pathogen, showing poor prognosis. *P. multocida* disease severity and the probability of transmission depends on the overall health status of an animal, stress, or environmental conditions like high temperature, humidity, poor nutrition, or work stress. This pathogen is characterized as non-motile, facultative anaerobic, coccobacillus, gram negative bacteria belonging to the family *Pasteurellaceae* ( Wilson and Ho 2013; Jilo et al. 2020; Mahrous et al. 2022).

According to WOAH, *P. multocida* is occasionally associated with zoonosis i.e., human acquisition of animal disease (Miyoshi et al. 2012; WOAH 2020). However multiple reports of human Pasteurellosis from the bite or lick of pet rabbits has been recorded ( Silberfein et al. 2006; Per et al. 2010).

Lack of proper animal disease management, poor hygiene conditions mainly in resource limited regions, delayed disease diagnosis, and no timely or effective treatment against *P. multocida* infection has shown to result in 35–40% morbidity rate with 23% mortality in six to eight months old rabbits (Premalatha et al. 2009). With this backdrop, we consider that rabbit immunization is the best method for effective protection and control strategy.

Inactivated vaccines are notably the most commonly used vaccines, which rely on a killed or inactivated pathogens to induce a protective immune response in host. Pathogen inactivation can be achieved either by chemical agents, heat, drying, or ionizing radiation like gamma rays, X-rays, or electron beam (Mostaan et al. 2020; Unger et al. 2022). The chemical inactivation relies on specific chemicals having bactericidal activity for example, formalin which is the earliest utilized chemical inactivator (Malinina et al. 1979), also commonly used in *P. multocida* vaccines production (El-Jakee et al. 2020). In addition, high concentrations of ferric chloride (FeCl_3_) are also used for production of chemical inactivated *P. multocida* vaccine. FeCl_3_ is known to generating free radicals to degrade bacterial DNA (Herath et al. 2010; Homayoon et al. 2018).

Despite long history of research and efforts to improve chemical inactivated vaccines, they are still associated with certain limitations. The current commercially available and experimental chemical inactivated vaccines against Pasteurellosis are known to be expensive, offers limited efficacy, require multiple doses, do not induce lifelong protection, and are also associated with undesirable side effects like inflammation at the site of injection (Mostaan et al. 2020).

As compared to chemical inactivation, radiation inactivation is an alternative technology in manufacturing killed or inactivated biological agents that can be used as safe vaccine candidates. Irradiated vaccines are found to overcome many limitations associated with chemical inactivated vaccine. Mounting evidence suggest that irradiation inactivated vaccines are cost effective, safe, and offer continued protection as compared to chemical inactivated vaccines (Bhatia and Pillai 2022; Unger et al. 2022). For example, immunization with gamma irradiated *P. multocida* vaccine adjuvanted with Montanide gel/01 has shown to be safe and offer 100% protection of chicken against fowl cholera as compared to the formalin inactivated fowl cholera vaccine that offered only 85% protection (Awoke et al. 2021).

Extensive experimental research in the field of irradiation technology has confirmed that exposure to specific doses of ionizing irradiation imparts inactivation because of the random structural damage to the bacterial genome. Previous studies suggest that the irradiation inactivated bacterial cell in addition to being non-replicative also retain residual metabolic and transcriptional activity under both in-vitro and in-vivo conditions (Ahmed et al. 2022; Unger et al. 2022). For example, findings from previous studies suggest that electron beam irradiated *Escherichia coli*, and gamma irradiated *Brucella melitensis*, *Mycobacterium bovis* and *Mannheimia haemolytica* are metabolically active but non-replicative after irradiation inactivation (Magnani et al. 2009; Secanella-Fandos et al. 2014; Hieke and Pillai 2018).

With this background, efforts should be made to develop new improved vaccine formulations against *P. multocida* infections. The present study aimed to use gamma irradiation as a tool to develop safe inactivated *P. multocida* vaccine candidates from field isolates and consequently to evaluate protection, serum antibody (IgG) levels and interferon gamma (IFN-γ) expression induced after rabbit immunization and bacterial challenge.

## Materials and methods

### Ethical declaration

The vaccine and challenge study on *P. multocida* infection in rabbits was performed in Giza at the National Research Centre (NRC) of Egypt. The experimental protocol was officially approved by the NRC animal ethics committee, under the trial registration number (TRN) no. 13602023, 13/5/2023. The study was performed in accordance with the Egyptian laws as well as the regulations of the NRC animal ethics committee. The laboratory rabbits in the study were cared for by a certified veterinarian and handled with acceptable practices and animal welfare standards.

### Bacterial isolation and identification

Bacterial isolation and identification were performed in accordance with accepted methods in microbiology (Songer and Post 2004). A total of 375 samples were collected from 250 infection suspected rabbits of different ages and sex that were clinically diagnosed to be suffering from *P. multocida* infection and from healthy rabbits suspected to be infected with *P. multocida*. The samples collected were 250 nasal swabs, 100 blood, and 25 lung swabs. The samples were immediately processed for bacteriological examination through in-vitro culturing on enriched growth media.

Samples were cultured on 5% sheep’s blood agar media and incubated at 37 °C for 24 h. To obtain a pure culture, a loop full of *Pasteurella* like bacterial colony from the agar media plate was re-cultured again on 5% sheep’s blood agar and incubated at 37 °C for 24 h. The pure bacterial culture was identified as *P. multocida* based on following criteria: 1) cellular morphology i.e., shape, color, size by gram staining, 2) biochemical identification, and 3) negative growth on MacConkey.

Bacterial cells stained with crystal violet and safranine dyes were examined microscopically at 100 X magnification (Smith and Hussey 2005). Biochemical tests including the catalase test, oxidase test, urease test, indole test, and nitrate reduction test were performed according to their standard protocols (Brink 2010; Reiner 2010; Shields and Cathcart 2010; Buxton 2011; MacWilliams 2012). Carbohydrate fermentation of these field isolates was evaluated by cultivating bacterial cells in carbohydrate fermentation media that contained peptone water with phenol red as pH indicator and 1% concentration of each carbohydrate i.e., either glucose, sucrose, sorbitol, mannitol, fructose, dulcitol, lactose, salicin, arabinose, and maltose. The inoculated carbohydrate fermentation media was incubated aerobically at 37 °C for 72 h. After incubation, color change and gas production (release of bubbles) were observed to indicate either positive or negative carbohydrate fermentation (Brogden 1980; Rahman et al. 2016).

### Enumeration and preparation of stock culture

For enumeration of field isolates of *P. multocida*, a loop full of *P. multocida* colony was suspended in 1 ml of nutrient broth and incubated at 37 °C for 24 h. Serial tenfold dilutions of bacterial suspension was made using nutrient broth, and 0.1 ml of each dilution was cultured on the Brain Hearth Infusion (BHI) agar media at 37 °C for 24 h. After characteristic colony formation, the number of viable cells were enumerated by evaluating the counting colony forming unit (CFU) in duplicate. Stock aliquots of confirmed field isolated of *P. multocida* were prepared in Phosphate Buffer Saline (PBS) to a final concentration of 1 × 10^10^ CFU/ml. Equal concentration of *P. multocida* in nutrient broth containing 80% sterile glycerin in eppendorf tubes were also stored at – 80 °C.

### Gamma irradiation of *P. multocida*

The irradiation procedure was carried out in the Gammacell-220 Excel (Cobalt-60 Canadian Radiator Facility) installed at the National Center for Radiation Research and Technology (NCRRT), Egyptian Atomic Energy Authority (EAEA), Cairo, Egypt. Fresh suspension culture of *P. multocida* was prepared in a 250 ml-conical flask containing 50 ml BHI broth and incubated at 37 °C for 24 h. Next day, the obtained bacteria cells were enumerated by the pour-plate counting method. Accordingly, the bacterial cell concentration was diluted to obtain 1 × 10^10^ CFU/ml by adding the equivalent volume of PBS. Then, the bacterial cell suspension was dispensed into sterile 15 ml-centrifuge tubes such that each tube contained 10 ml.

The bacterial cells were collected as a pellet in each tube and washed twice by PBS. Then, the bacterial pellet was re-suspended in 10 ml PBS as a final volume. The dose rate at the time of the experiment equaled 0.89 KGy/hour. Each triplicate was packed with ice into a cooling bag. To detect the optimum radiation dose required for preparation of gamma irradiated *P. multocida* vaccine, the bacterial cells were exposed to six different irradiation doses as 0, 4, 6, 8, 10, and 12 KGy. The decrease of the CFU count, due to the irradiation, was determined by inoculating 1 ml of each bacterial cell suspension on a BHI agar plate and incubated at 37 °C for 24 h. The optimum radiation dose was selected based on the lowest amount of radiation rendering *P. multocida* non-replicating (Aquino et al. 2005; Hammad et al. 2010).

### Preparation of different gamma irradiated vaccine formulations and bacterial challenge

All the three types of gamma irradiated *P. multocida* vaccine formulations were prepared at a dose of 2 × 10^9^ cells/ml in PBS i.e., the single vaccination dose. For preparation of different irradiated vaccine formulations, three tubes of each bacterial formulation were exposed to the optimal gamma ray dose under colling. The liquid formulations of bacteria were prepared by re-suspending the bacterial pellet in 10 ml PBS as a final volume. While the lyophilized formulations of bacteria were prepared by re-suspending the bacterial pellet in 1 ml BHI broth with or without 10% (W/V) Trehalose before lyophilization. For preparing activated lyophilized trehalose irradiated vaccine formulation, the lyophilized trehalose irradiated bacterial solution was further incubated at 37 °C for overnight to facilitate the activation of residual metabolic activity in non-replicating *P. multocida* cultures, then the bacterial cells were centrifuged, the pellet washed twice in PBS and re-suspended in PBS.

For preparation of challenge inoculum, *P. multocida* was cultivated on BHI agar plate at 37 °C for 24 h under aerobic condition. The bacterial colonies were harvested in PBS, washed twice, and the cell pellet was diluted in 1 ml PBS to a final concentration of 3.6^10^ CFU/ml i.e., the signal challenge dose according to Lu and Pakes (Lu and Pakes 1981).

### Animal handling, immunization, and infection challenge

White New Zealand rabbit confirmed to be free of pathogens and without any history of vaccination, were purchased from the Animal Production Research Institute, New Zealand and were transferred to vivarium. Rabbits between seven to eight weeks old and barrier-bred, were allowed a one-week period to acclimatize to their new environment. The rabbits were kept individually in stainless steel cages, wherein the slatted bottoms had no bedding. All rabbits were given ad libitum access to fresh tap water and balanced commercial feed.

**Fig. 1:**
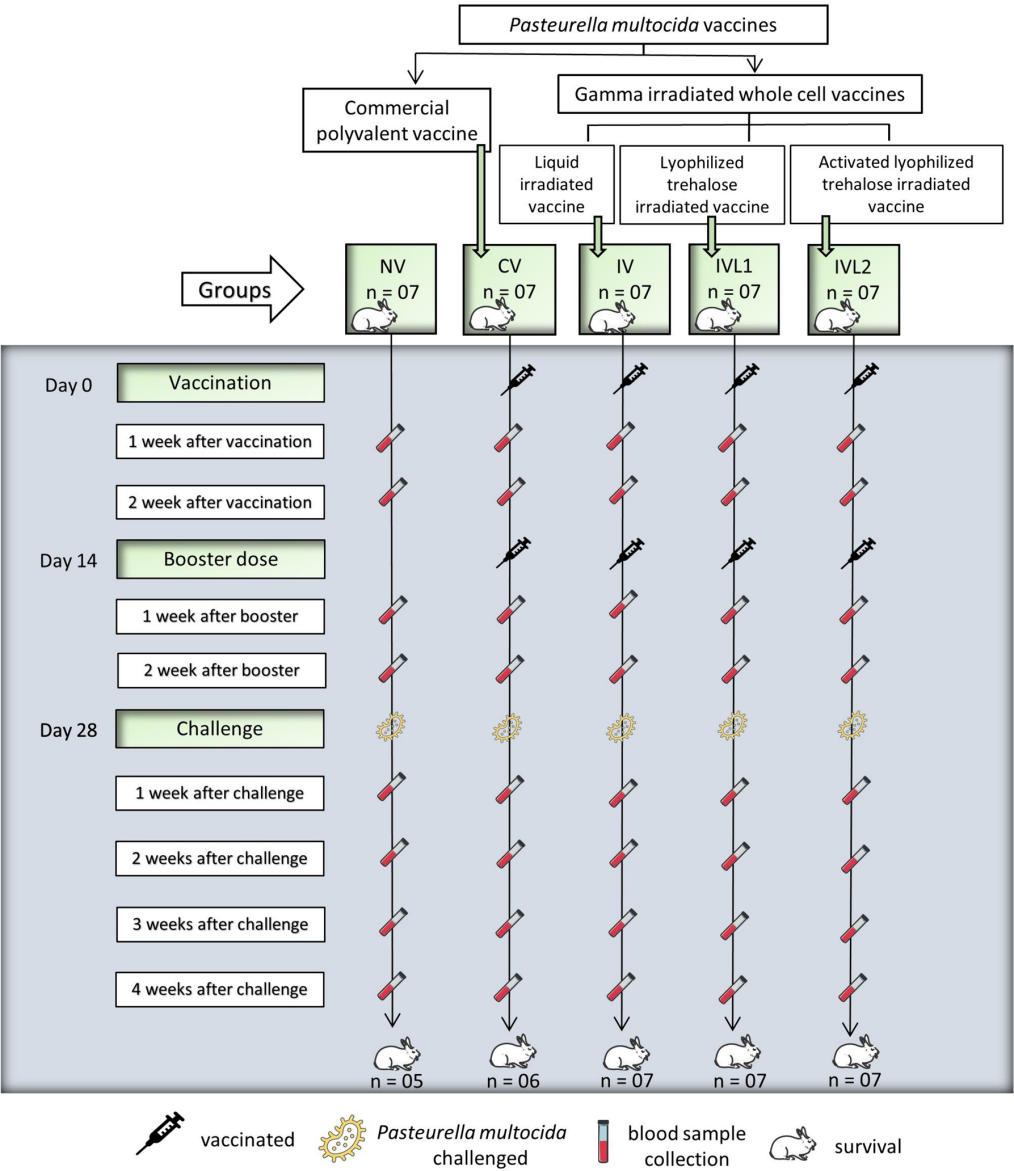
Schematic diagram for animal immunization and experimental design for clinical trial of γ-irradiated *P. multocida* vaccine in rabbits. Source: The illustration was generated using Servier Medical Art, Licensed under a Creative Common Attribute 3.0 unported license 1

The schematic illustration of the performed experimental setup and sampling schedule is shown in Fig. 1. Upon the rabbit’s arrival at vivarium, they were divided into five separate groups, each group having seven rabbits. The five groups were individually tagged with a specific code to identify them as: (1) the non-vaccinated control i.e., NV. (2) vaccinated with commercial *P. multocida* vaccine i.e., CV. (3) vaccinated with liquid irradiated vaccine formulation with no trehalose i.e., IV. (4) vaccinated with lyophilized trehalose irradiated vaccine i.e., IVL1. (5) vaccinated with activated lyophilized trehalose irradiated vaccine i.e., IVL2.

The chemical inactivated commercial vaccine used for CV group was a local polyvalent rabbit Pasteurellosis vaccine produced at Veterinary Serum and Vaccine Research Institute, Cairo, Egypt, the vaccination dose was according to production instructions.

For immunization, the IV, IVL1, and IVL2 subcutaneously received 2 × 10^9^ cells/ml of first vaccine dose on day 0, followed by a similar concentration of booster dose administered on day 14 (two weeks after first dose). Likewise, rabbits in challenge only group i.e., NV group received the same volume of PBS two times instead of vaccine. Two weeks after booster dose (day 28), rabbits of all five groups were challenged with *P. multocida* bacteria cells of field isolates. The challenge dose of 1 ml (3.6^10^ CFU/ml) was administered via the subcutaneous route. The immune response and growth weight were assessed in five rabbits per group. The body weight was measured at day zero, before the bacterial challenge and two weeks after bacterial challenge.

### Sampling and serum preparation

Whole blood samples were obtained from all rabbits. The schematic representation of sampling schedule is shown in Fig. 1. After sampling the blood was immediately processed for serum preparation. The blood collection and serum preparation was done according to the recommended standard protocol (Greenfield 2018) from the tip of marginal ear vein using a 19 – 23 gauge butterfly needle. Blood samples were allowed to form clots, followed by the removal of serum from the clot by centrifugation. The serum samples were transferred to sterile tubes. Aliquots of serum samples were made, labeled with animal code and the collection date. The aliquots were kept at –20 °C until further processing.

### Measuring IgG expression

The serum samples of rabbits from each group were processed through an optimized in-house indirect ELISA protocol. All samples including the positive serum, negative serum, and PBS-blank were measured in duplicates. The 96-well polystyrene microtiter flat-bottom plates were coated overnight with 1:16 diluted antigen (sonicated *P. multicida* of concentration 2 × 10^9^ CFU/ml) in 50 mM carbonate buffer, pH 9.6.

The sonication was achieved within 15 min at 35% power through a cell-disrupter having microtip probe. The antigen coated plate was incubated overnight at 4 °C. Blocking of free sites was carried out using 3% gelatin in a coating buffer for 1 h at room temperature. After incubation the wells were washed three times using wash buffer (0.01 M TBS containing 0.05% Tween 20, pH 7.4).

Sera diluted two times in 0.01 M TBS containing 0.5% gelatin (pH 7.4) was added and incubated for 1 h at room temperature. After incubation, the wells were washed three times with a wash buffer. After washing, plates were incubated with 100 µl of 1:10000 diluted commercially available rabbit IgG (heavy and light chain specific) horseradish peroxidase (HRP) conjugated polyclonal antibody (Bethyl Laboratories Inc., USA; Cat No. A120-101P). The plate was incubated at 37 °C for 1 h. After incubation the wells were washed three times followed by addition of 100 µl of O-Phenylenediamine (OPD) chromogen substrate solution (Sigma Aldrich) to all wells.

The plate was incubation at 37 °C for 15 min in dark and color change was noted. Immediately after incubation, 25 µl of 1.25 mol sulfuric acid (H_2_SO_4_) stop solution was added to each well. The optical density (OD) for each sample was recorded by measuring the absorbance at 450 nm wavelength using microplate reader (BioTek ELX800, Gen5 2.00) (El-Shershaby et al. 2023).

### Measuring IFN-γ expression

A commercially available IFN-γ ELISA kit (Cat No. MCA5638KZZ, Bio-Rad) was used. The serum samples of five rabbits from each group were processed through a sandwich ELISA protocol which was followed as described in the kit manual. All samples including the positive serum, negative serum, and PBS-blank were measured in duplicates.

### Statistical data analysis

GraphPad Prism 8 software was used to statistically analyze the ELISA data. Two-Way ANOVA test and Tukey multiple comparisons were performed to analyze the antibody titers and IFN-γ titers. The columns represent the mean values and error bars were used to indicate the standard deviation (SD). The data was recorded at the level of significance or P values of 0.05 at 95% confidence intervals. The P value higher than 0.05 (*P* > 0.05) were considered statistically non-significant, *P* ≤ 0.05 were denoted by one asterisk and considered slightly significant, *P* ≤ 0.01 were denoted by two asterisk and considered moderately significant, and *P* ≤ 0.001 were denoted by three asterisk and considered highly significant.

## Results

### Characteristics of* Pasteurella multocida* field isolates

Cultural, morphological, gram staining, and biochemical examination facilitated the selection and characterization of rabbit *P. multocida*. The *P. multocida* from rabbits were found to grow well on 5% sheep’s blood agar media and BHI agar media. Pure colonies on blood agar media resulted in no hemolysis of red blood cells. Based on morphological characteristics the pure colonies were small, round, gray in color, and mucoid. The pure culture of *P. multocida* retained safranin dye and appeared pink under microscope and therefore the pure bacterial culture was identified as gram-negative. On MacConkey agar the *P. multocida* showed no growth. The results of the biochemical tests are shown in Table 1. The biochemical examination revealed that the pure bacterial culture of rabbit *P. multocida* was catalase, oxidase, indole, and nitrate reductase test positive, but showed negative urease test result. Carbohydrate fermentation results revealed that pure *P. multocida* isolates were able to ferment glucose, sucrose, mannitol, and fructose. But sorbitol, dulcitol, lactose, salicin, arabinose, and maltose were not fermented. These findings were similar to previously reported characteristics of rabbit *P. multocida* (Brogden 1980; Rahman et al. 2016; Songer and Post 2004). The obtained positive samples for *P. multocida* were 196 out of 275 samples.

**Table 1:** Biochemical tests and their results against *P. multocida* field isolates from rabbits

Biochemical Tests		Results
Catalase		+
Oxidase		+
Urease		-
Indole		+
Nitrate reduction		+
Carbohydrate fermentation	Glucose	+
	Sucrose	+
	Mannitol	+
	Fructose	+
	Sorbitol	-
	Dulcitol	-
	Lactose	-
	Salicin	-
	Arabinose	-
	Maltose	-

Positive test ( +), negative test (-)

### Gamma irradiated* Pasteurella multocida* vaccine

All the different bacterial suspensions in PBS were exposed to a range of gamma ray doses i.e., 4 kGy, 6 KGy, 8 KGy, 10 KGy, and 12 KGy to determine the optimal dose required to stop replication of *P. multocida*. The irradiated *P. multocida* samples from each irradiation experiment when re-cultured on BHI agar plate showed that the lowest irradiation dose that causes loss of replication was achieved with 10 KGy.

### Serum antibody response

**Fig. 2:**
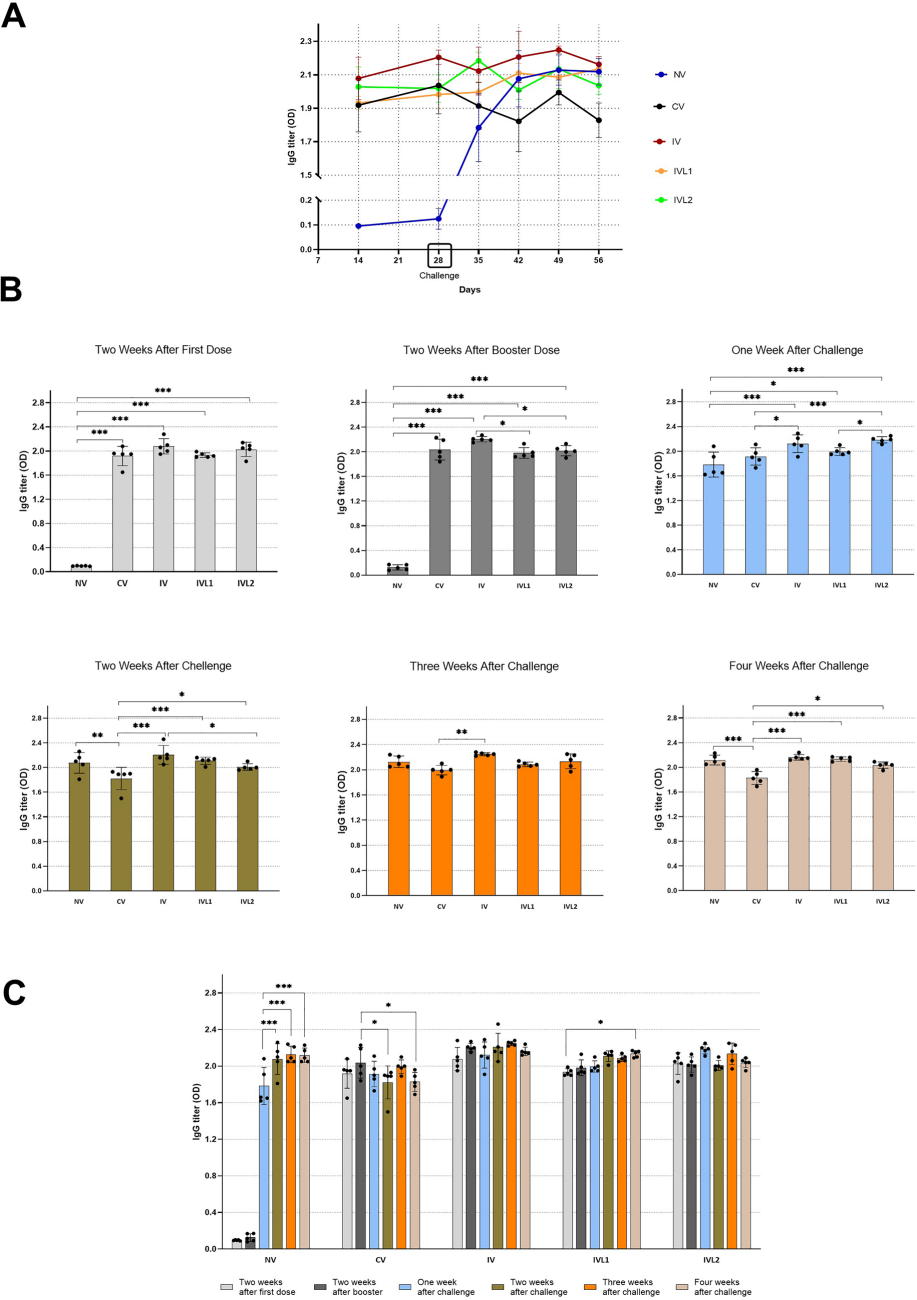
Serum IgG antibody titer against *P. multocida* infection in vaccinated and nonvaccinated rabbits. **A** IgG titer measured at 450 nm for NV, CV, IV, IVL1 and IVL2 groups. **B** The significant difference increased compared to the control at ***P ≤ 0.001. **C** the antibodies titer for each group. Significance difference indicated as *P < 0.05, **P <0.01, ***P < 0.001

The OD values measured at 450 nm and the mean IgG titers can be seen in Table 2 and Fig. 2. The peak mean of serum IgG titer for commercial vaccine (CV) was recorded at two weeks after booster dose which was OD 2.036, however this titer was significantly reduced with passage of time. The IV and IVL2 immunized groups maintained it IgG titer till day 56, whereas the IVL1 showed a slight significant increase in IgG titer from day 14 to day 56. The peak mean of serum IgG titer for liquid irradiated vaccine (IV) was observed at two weeks after booster dose which was OD 2.204 and also at three weeks after challenge which was OD 2.248. The peak mean of serum IgG titer for lyophilized trehalose irradiated vaccine (IVL1) was observed at two weeks after challenge which was OD 2.110 and also at four weeks after challenge which was OD 2.134. The peak mean of serum IgG titer for activated lyophilized trehalose irradiated vaccine (IVL2) was observed at one weeks after challenge which was OD 2.184 and three weeks after challenge which was OD 2.134. The peak mean of IgG titer for control was recorded on day 49 i.e., three weeks after challenge which was OD 2.128.

**Table 2:** ELISA data measured at 450 nm for rabbits’ serum IgG levels

Control						
Weeks	NV^A^	NV^B^	NV^C^	NV^D^	NV^E^	Mean
Two weeks after first dose	0.101	0.1	0.095	0.089	0.093	0.1966
Two weeks after booster dose	0.168	0.17	0.091	0.079	0.117	0.125
One week after challenge	1.91	2.08	1.66	1.65	1.62	1.784
Two weeks after challenge	2.16	2.05	1.81	2.26	2.1	2.076
Three weeks after challenge	2.21	2.05	2.09	2.24	2.05	2.128
Four weeks after challenge	2.17	2.05	2.23	2.09	2.05	2.118
Group 1 – Local Commercial Polyvalent Vaccine						
Weeks	CV^A^	CV^B^	CV^c^	CV^D^	CV^E^	Mean
Two weeks after first dose	1.96	1.65	1.95	2.08	1.95	1.918
Two weeks after booster dose	2.23	1.92	1.84	2.19	2	2.036
One week after challenge	1.73	1.97	1.9	2.11	1.86	1.914
Two weeks after challenge	1.89	1.93	1.5	1.9	1.89	1.822
Three weeks after challenge	2	2	1.89	1.98	2.1	1.994
Four weeks after challenge	1.96	1.82	1.78	1.69	1.89	1.828
Group 2 – Liquid Irradiated Vaccine Without Trehalose						
Weeks	IV^A^	IV^B^	IV^c^	IV^D^	IV^E^	Mean
Two weeks after first dose	1.95	2.06	2.28	2.1	2	2.078
Two weeks after booster dose	2.19	2.15	2.21	2.27	2.2	2.204
One week after challenge	1.91	2.09	2.21	2.29	2.11	2.122
Two weeks after challenge	2.05	2.46	2.21	2.16	2.15	2.206
Three weeks after challenge	2.23	2.26	2.28	2.22	2.25	2.248
Four weeks after challenge	2.12	2.24	2.14	2.16	2.15	2.162
Group 3—Lyophilized Trehalose Irradiated Vaccine						
Weeks	IVL1^A^	IVL1^B^	IVL1^c^	IVL1^D^	IVL1^E^	Mean
Two weeks after first dose	1.99	1.95	1.9	1.9	1.92	1.932
Two weeks after booster dose	1.92	1.93	2.13	1.94	1.99	1.982
One week after challenge	1.95	2.1	1.98	1.95	2	1.996
Two weeks after challenge	2.01	2.16	2.12	2.14	2.12	2.11
Three weeks after challenge	2.1	2.14	2.06	2.05	2.08	2.086
Four weeks after challenge	2.17	2.15	2.1	2.09	2.16	2.134
Group 4 – Activated Lyophilized Trehalose Irradiated Vaccine						
Weeks	IVL2^A^	IVL2^B^	IVL2^c^	IVL2^D^	IVL2^E^	Mean
Two weeks after first dose	2.09	1.83	2.03	2.14	2.05	2.028
Two weeks after booster dose	2.01	1.9	2.05	2.13	2	2.018
One week after challenge	2.17	2.11	2.25	2.19	2.2	2.184
Two weeks after challenge	2.1	1.96	2	1.98	2	2.008
Three weeks after challenge	2.06	2.25	2.23	1.97	2.16	2.134
Four weeks after challenge	2.09	2.04	2.04	1.95	2.06	2.036

The results showed that two weeks after the first vaccine dose (day 14), the levels of serum IgG titers were significantly increased (***P ≤ 0.001) in the all the vaccine treated groups i.e., CV, IV, IVL1 and IVL2 as compared to the non-vaccinated control. Also, on day 14 the commercial polyvalent vaccine and all three gamma irradiated vaccine formulations resulted in augmenting almost similar serum IgG levels. Two weeks after booster dose (day 28), the IgG titers within each individual vaccine treated group had neither significantly increase nor decrease as compared to the day 14 however, across the different vaccine treated groups the liquid irradiated vaccine formulation with no trehalose (IV) showed significantly higher IgG titer (*P ≤ 0.05) than both lyophilized-trehalose irradiated vaccine formulations (IVL1 and IVL2). Furthermore, on day 28 no significance difference was recorded between the liquid irradiated vaccine formulation (IV) and the commercial polyvalent vaccine (CV).

One week after challenge (day 35), all three irradiated vaccine formulations showed statistically higher IgG titers compared to the commercial vaccine (CV) and control. The commercial vaccine recorded no significant difference with control. In addition, the activated lyophilized-trehalose irradiated vaccine (IVL2) as compared to the other lyophilized-trehalose irradiated vaccine (IVL1) showed a statistically significant of *P equals to 0.04. The non-vaccinated control experienced a robust seroconversion that was statistically significant (***P ≤ 0.001) as compared to the before challenge, as well as with each passing week in control the IgG titers kept on increasing significantly (***P ≤ 0.001) as compared to one week after challenge.

Two weeks after challenge (day 42), in the commercial vaccine (CV) IgG titers reduced, that resulted in it to have significantly lower IgG titer as compared to control and all the gamma irradiated vaccine formulations. Simultaneously, on day 42 the liquid irradiated vaccine recorded a significantly higher IgG titers as compared to the activated lyophilized trehalose irradiated vaccine (*P = 0.03). Three weeks after challenge (day 49), only the liquid irradiated vaccine (IV) remained to produce higher IgG titers as compared to the commercial vaccine (**P = 0.002).

On day 49, no significant difference was recorded between the control and the different gamma irradiated vaccine formulations. At four weeks after challenge (day 56), all the irradiated vaccine formulations showed to have maintained a significantly higher IgG antibody titers as compared to the commercial vaccine.

### Interferon gamma response

**Fig. 3:**
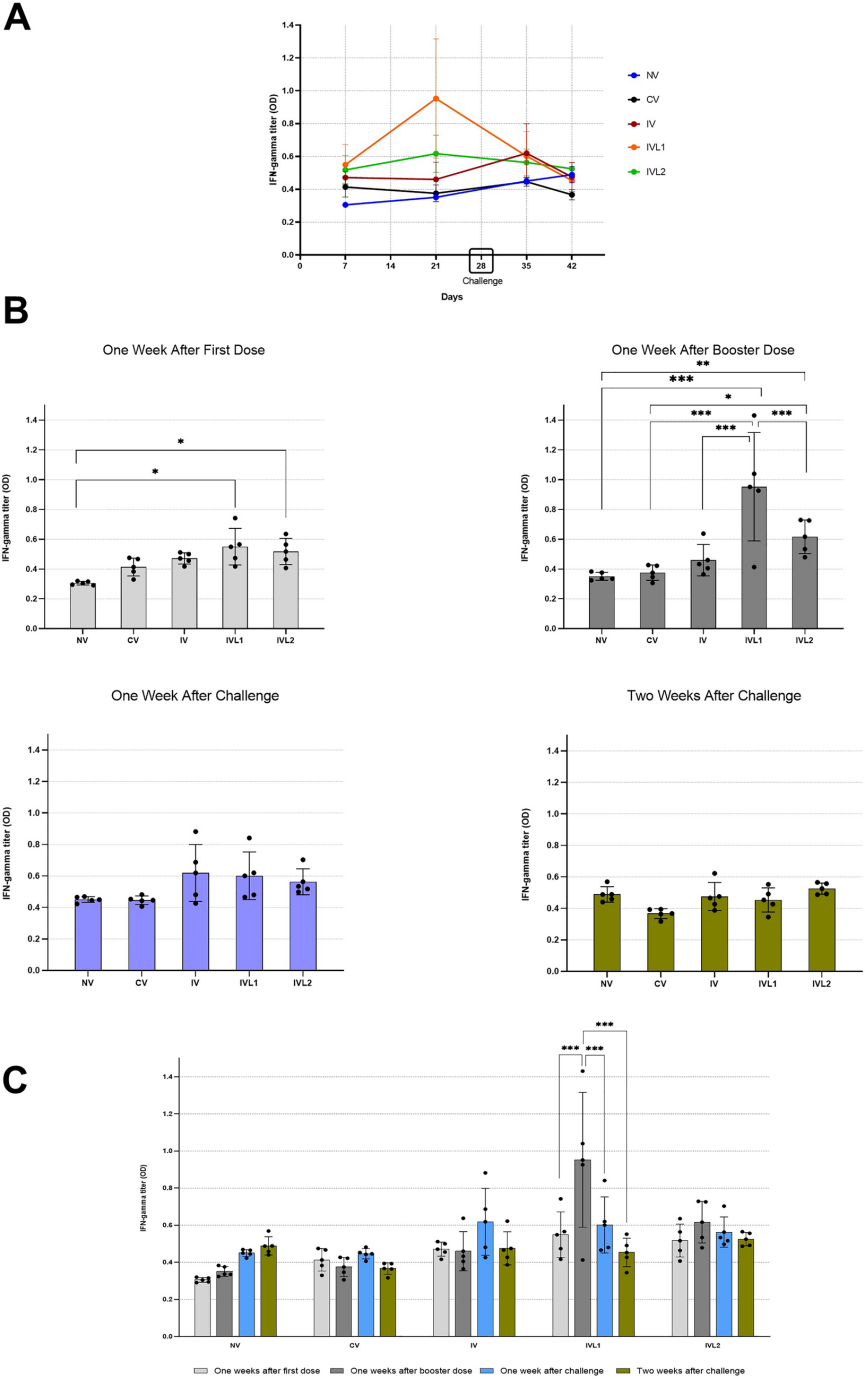
Interferon gamma (IFN-γ) levels against *P. multocida* infection in rabbits. **A** IFN-γ levels measured at 450 nm for NV, CV, IV, IVL1 and IVL2. **B** Comparison of serum IFN-γ expression level among different groups. Significance difference indicated as *P ≤ 0.05, **P ≤ 0.01, ***P ≤ 0.001. **C** Comparison of serum IFN-γ expression level within individual groups. Significance difference indicated as *P ≤ 0.05, **P ≤ 0.01, ***P ≤ 0.001

The IFN-γ serum titers measured by sandwich ELISA are shown in Table 3 and Fig. 3. The results of OD values measured at 450 nm showed that one week after first dose (day 7), a statistically significant increase was observed in IFN-γ titers by lyophilized-trehalose irradiated vaccine (IVL1) (*P = 0.01) and the activated lyophilized-trehalose irradiated vaccine (IVL2) (*P = 0.04) as compared to the control, commercial vaccine (CV), and liquid irradiated vaccine with no trehalose (IV). One week after booster dose (day 21), the lyophilized-trehalose irradiated vaccine (IVL1) showed significant increase (***P < 0.001) in IFN-γ titers as compared to all other groups. Furthermore, on day 21 the activated lyophilized-trehalose irradiated vaccine (IVL2) even though was significantly (***P < 0.001) at a lower IFN-γ titer as compared to IVL1, but still its IFN-γ titer was statistically higher than the commercial vaccine (CV) (*P = 0.01) and the control (**P = 0.004). At one week after challenge (day 35) and two weeks after challenge (day 42), the control and all vaccine treated groups showed no significant difference in their IFN-γ titers. Within each individual group i.e., the NV, CV, IV, and IVL2 showed no statistical significance with each passing week. However, the lyophilized trehalose irradiated vaccine (IVL1) showed peak mean of IFN-γ titer at one week after booster dose (day 21) which was statistically significant (***P < 0.001) as compared to day 7, day 35, and day 42 in IVL1.

**Table 3:** ELISA data measured at 450 nm for rabbits’ serum IFN-γ levels

Control						
Weeks	NV^A^	NV^B^	NV^C^	NV^D^	NV^E^	Mean
One weeks after first dose	0.295	0.316	0.32	0.291	0.305	0.3054
One weeks after booster dose	0.383	0.376	0.337	0.324	0.335	0.351
One week after challenge	0.469	0.445	0.423	0.465	0.45	0.4504
Two weeks after challenge	0.459	0.44	0.488	0.569	0.489	0.489
Group 1 – Local Commercial Polyvalent Vaccine						
Weeks	CV^A^	CV^B^	CV^C^	CV^D^	CV^E^	Mean
One weeks after first dose	0.475	0.33	0.383	0.467	0.413	0.4136
One weeks after booster dose	0.427	0.422	0.347	0.306	0.375	0.3754
One week after challenge	0.482	0.406	0.443	0.453	0.446	0.446
Two weeks after challenge	0.393	0.317	0.394	0.364	0.367	0.367
Group 2 – Liquid Irradiated Vaccine Without Trehalose						
Weeks	IV^A^	IV^B^	IV^C^	IV^D^	IV^E^	Mean
One weeks after first dose	0.456	0.417	0.512	0.5	0.471	0.4712
One weeks after booster dose	0.638	0.433	0.405	0.365	0.46	0.4602
One week after challenge	0.481	0.687	0.882	0.426	0.619	0.619
Two weeks after challenge	0.466	0.387	0.427	0.622	0.475	0.4754
Group 3—Lyophilized Trehalose Irradiated Vaccine						
Weeks	IVL1^A^	IVL1^B^	IVL1^C^	IVL1^D^	IVL1^E^	Mean
One weeks after first dose	0.742	0.474	0.566	0.417	0.549	0.5496
One weeks after booster dose	0.413	1.431	1.04	0.926	0.952	0.9524
One week after challenge	0.62	0.841	0.466	0.48	0.601	0.6016
Two weeks after challenge	0.491	0.552	0.345	0.427	0.453	0.4536
Group 4—Activated Lyophilized Trehalose Irradiated Vaccine						
Weeks	IVL2^A^	IVL2^B^	IVL2^C^	IVL2^D^	IVL^E^	Mean
One weeks after first dose	0.464	0.564	0.407	0.636	0.517	0.5176
One weeks after booster dose	0.729	0.726	0.479	0.534	0.617	0.617
One week after challenge	0.703	0.517	0.498	0.534	0.563	0.563
Two weeks after challenge	0.565	0.492	0.487	0.557	0.525	0.5252

### Vaccine safety

No death or side effect was recorded in rabbits vaccinated with irradiated *P. multocida* vaccine compared to the commercial vaccine which caused the death of one rabbit out of seven before the bacterial challenge After bacterial challenge, two deaths were recorded in the control group (Fig. 1).

### Immune protection with vaccines

**Fig. 4:**
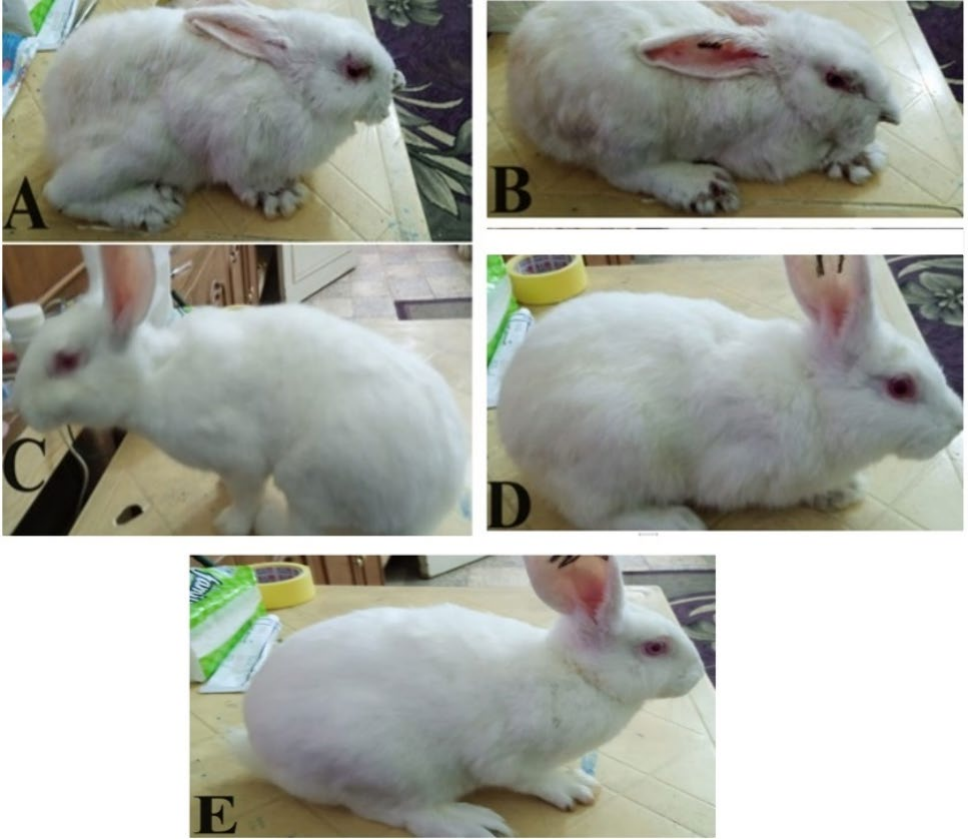
Illustres the rabbits healthy status after bacterial challenge, **A** (control group, all five rabbits), **B** (CV, two out of six rabbits), **C** (IV, all rabbits), **D** (IVL1, all rabbits) and **E** (IVL2, all rabbits)

Immunization of rabbits twice through the subcutaneous route with the liquid irradiated non-trehalose vaccine, the lyophilized trehalose irradiated vaccine, and the activated lyophilized trehalose irradiated vaccine resulted in no clinical signs of sickness or injection site reactions in rabbits. The immunization with commercial polyvalent *P. multocida* vaccine resulted in skin inflammation at the site of vaccination injection. Thus, the three irradiated vaccine formulations were regarded as safe to be administered subcutaneously in rabbits. Moreover, commercial-vaccinated rabbits (CV) suffered from mange infection compared to rabbits immunized with irradiated vaccines (IV, IVL1, and IVL2) (Fig. 4).

**Fig. 5:**
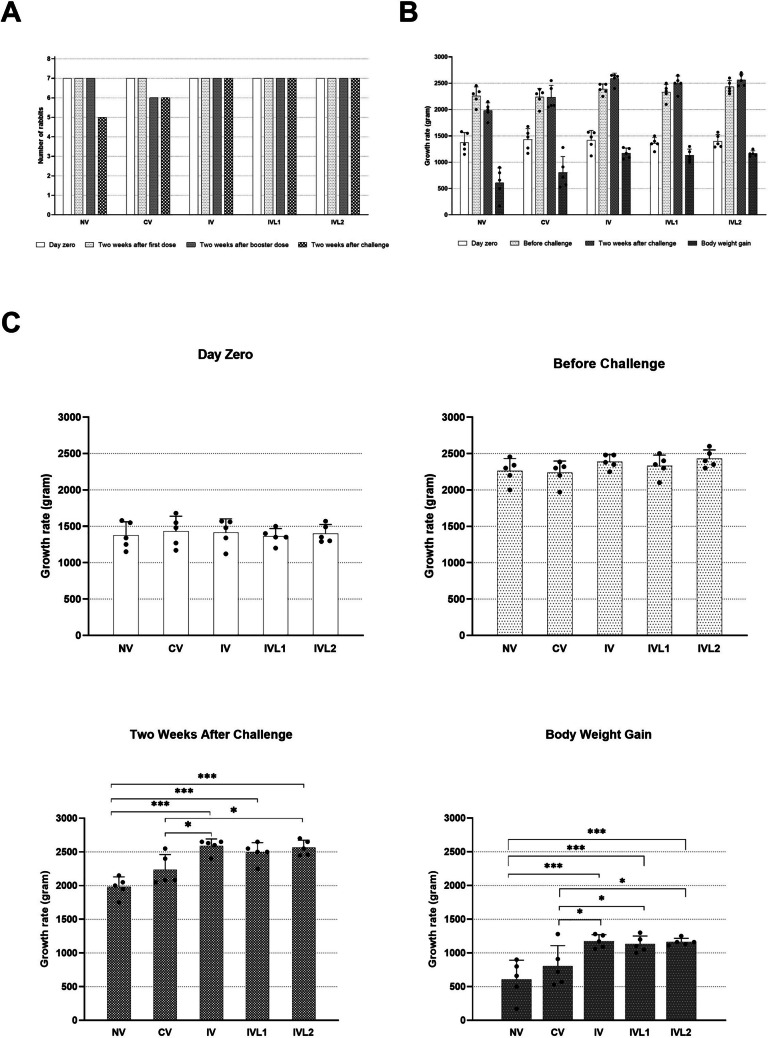
**A** Mortality rate, **B** and **C** Comparison of the growth rate between the different groups. Significance difference indicated as *P ≤ 0.05, **P ≤ 0.01, ***P ≤ 0.001

The rabbits vaccinated with the different formulations of irradiated vaccine showed increases in growth rate compared to the commercial vaccine animals which recorded less growth weight (Table 4). The results of body weight gain from day zero to two weeks after bacterial challenge showed significant difference between the control and the vaccinated groups by irradiated vaccine and also between the vaccinated group by commercial vaccine and the vaccinated groups by irradiated vaccine. The difference in body weight gain between the control group and the commercially vaccinated group was non-significant (Fig. 5). After the bacterial challenge, no clinical symptoms were observed in the irradiated vaccine rabbits while the commercial vaccinated rabbits showed nose snuffle in some rabbits and loss in body weight.

**Table 4:** The growth rate/gram between the different groups

Control						
Weeks	NV^A^	NV^B^	NV^D^	NV^C^	NV^E^	Mean
At day zero	1150	1250	1340	1580	1550	1374
Before the bacterial challenge	2200	2340	2455	2000	2300	2259
Two weeks after bacterial challenge	1950	2150	2000	1750	2050	1980
Body weight gain	800	900	660	170	500	606
Group 1 – Local Commercial Polyvalent Vaccine						
Weeks	CV^A^	CV^B^	CV^C^	CV^D^	CV^E^	Mean
At day zero	1170	1480	1550	1680	1270	1430
Before the bacterial challenge	2385	1970	2200	2300	2320	2235
Two weeks after bacterial challenge	2080	2050	2080	2400	2550	2232
Body weight gain	910	570	530	720	1280	802
Group 2 – Liquid Irradiated Vaccine Without Trehalose						
Weeks	IV^A^	IV^B^	IV^C^	IV^D^	IV^E^	Mean
At day zero	1120	1560	1480	1570	1340	1414
Before the bacterial challenge	2250	2480	2350	2480	2380	2388
Two weeks after bacterial challenge	2400	2650	2650	2630	2600	2586
Body weight gain	1280	1090	1170	1060	1260	1172
Group 3—Lyophilized Trehalose Irradiated Vaccine						
Weeks	IVL1^A^	IVL1^B^	IVL1^c^	IVL1^D^	IVL1^E^	Mean
At day zero	1400	1350	1350	1500	1200	1360
Before the bacterial challenge	2400	2500	2300	2350	2100	2330
Two weeks after bacterial challenge	2500	2650	2550	2500	2250	2490
Body weight gain	1100	1300	1200	1000	1050	1130
Group 4 – Activated Lyophilized Trehalose Irradiated Vaccine						
Weeks	IVL2^A^	IVL2^B^	IVL2^C^	IVL2^D^	IVL2^E^	Mean
At day zero	1300	1350	1570	1490	1290	1400
Before the bacterial challenge	2400	2350	2600	2500	2300	2430
Two weeks after bacterial challenge	2550	2460	2700	2650	2450	2562
Body weight gain	1250	1110	1130	1160	1160	1162

## Discussion

Vaccine and infection studies against *P. multocida* has been primarily focused on controlling animal diseases (Wilson and Ho 2013) because of relatively low incidence of *P. multocida* infection in humans (WOAH 2020). The development and evaluation of safe, cost effective, immunogenic, and protective animal vaccines have been challenging. Despite considerable efforts and diverse experimental animal models, the available commercial and experimental vaccines do not provide complete immunity as well as are not effective in clearing bacterial infection (Wilson and Ho 2013). Currently there is a need for a safe vaccine that can provide persistent immune protection with neutralization of challenge infection, to help mitigate rate of bacterial diseases in rabbit production, which is contributing to substantial economic loss especially in resource limited countries. Despite locally available chemical inactivated polyvalent vaccines Pasteurellosis still remains a significant bacterial disease in rabbits. Thereby, keeping in view the high susceptibility rate of disease in rabbits (El-Ghany and Wafaa 2023) the present study demonstrates the production of three different formulations of gamma irradiated *P. multocida* vaccine from rabbit field isolates and evaluation of immunological changes caused by these irradiated vaccine formulations or local commercial polyvalent vaccine in rabbits.

In our study, gamma irradiation dose of 10 KGy inactivated the rabbit *P. multocida* as confirmed by the subculturing on BHI. To our knowledge there are no available reports on using gamma irradiated *P. multocida* vaccine for immunization in rabbits against *P. multocida* infection. We present that the irradiated *P. multocida* administered subcutaneously in either liquid or lyophilized with or without Trehalose is safe and protective, indicating its potential use for immunization of rabbits. A previous study based on using liquid gamma irradiated avian *P. multocida* Montanide gel/01 adjuvanted vaccine for immunization of chickens was also reported to be safe and protective against fowl cholera (Awoke et al. 2021).

Irradiated vaccines are found to induce humoral immune response, making these vaccine formulations highly effective. In this study, different gamma irradiated vaccine formulations including liquid irradiated vaccine, lyophilized-trehalose irradiated vaccine, and activated lyophilized trehalose irradiated vaccine all induced serum IgG response upon immunization as well as protect rabbits against sever infection complications after bacterial challenge. Immunization with commercial polyvalent vaccine even though also induced antibody response but indicated a significant decline in serum IgG titer with each passing week after challenge as compared to the irradiated vaccines. We suggest that the preserved immunogenic structures of protein antigens after irradiation (Mahmoud et al. 2016) might have contributed to continued humoral response in irradiated vaccine treated groups (IV, IVL1, and IVL2).

IFN-γ qualifies as a critical regulating cytokine that orchestrate diverse biological functions including augment inflammation, development of Th1 over Th2 cells, augment bactericidal activity of macrophage, directing isotype switch of B cells, and indirectly affect chemokine production. Produced by innate-like lymphocyte population and adaptive immune cells (Natural Killer cells, T helper 1 cells, CD4 + T-cell and CD8 + T- cells), IFN-γ plays a pivotal role in immunity against endogenous as well as exogenous agents (Billiau and Matthys 2009). Early findings suggest that IFN-γ regulates the pathomechanism of *P. multocida* toxin (PMT) associated pneumonia in lungs. A study demonstrated that IFN-γ deficient mice when challenged with recombinant PMT have significantly higher survival rate and reduced lung lesions (Xiao et al. 2023). These observations show that elevated IFN-γ expression might be responsible for severity of infection. In this study, we observed that immunization with liquid irradiated vaccine and commercial vaccine and subsequent challenge does not induce a significant increase in IFN-γ expression as compared to control. In contrast, both lyophilized trehalose vaccine formulations presented a significant rise in IFN-γ expression after immunization as compared to control, however, after challenge infection, the IFN-γ expression reduced back to normal physiological levels. This implies that among irradiated vaccines IFN-γ expression is maintained at a normal level, most notably by liquid irradiated vaccine, and promotes development of a weak infection that is not lethal to vaccinated animals but effective enough to provide immune protection.

Concerning vaccine efficacy, this study reports that subcutaneous immunization with irradiated vaccines using either liquid or lyophilized preparations with or without Trehalose results in 100% protection against infection challenge as well as is devoid of any vaccination side effects, wherein the rabbits maintain its normal physiological state. On the contrary, our finding showed that the current commercially available polyvalent vaccine causes skin inflammation at injection site in rabbits and can also be slightly lethal because immunization with it resulted in death of one rabbit out of seven. The increase in body weight of the groups vaccinated by irradiated vaccine added more advantages for irradiation vaccine compared to the commercial polyvalent vaccine (Table 4 and Fig. 5).

## Conclusion

This study investigated the role of irradiation in vaccine production as well as the protection developed after immunization and challenge infection in rabbits against *P. multocida*. From this study observations, we infer that gamma irradiation inactivated *P. multocida* vaccines can lead to a robust humoral immune response and is a safer alternative for effective and continued protection against rabbits’ infection associated with *P. multocida* as compared to chemically inactivated commercial vaccine. Safe animal vaccine and immunization strategies can contribute to cost saving, healthier life, and sustainable communities. We consider that this information will facilitate and promote the investment of research resources in evaluation of other whole cell inactivated vaccine formulations, acceptance of using irradiation as a tool for developing vaccines, and its approval for national use.

## Data Availability

The data sets generated during the current study are available from the corresponding author upon reasonable request.
